# Extracellular Regulated Kinase 1/2 Signaling Is a Critical Regulator of Interleukin-1β-Mediated Astrocyte Tissue Inhibitor of Metalloproteinase-1 Expression

**DOI:** 10.1371/journal.pone.0056891

**Published:** 2013-02-14

**Authors:** Jerel Fields, Irma E. Cisneros, Kathleen Borgmann, Anuja Ghorpade

**Affiliations:** Department of Cell Biology and Anatomy, University of North Texas Health Science Center, Fort Worth, Texas, United States of America; University of Torino, Italy

## Abstract

Astrocytes are essential for proper central nervous system (CNS) function and are intricately involved in neuroinflammation. Despite evidence that immune-activated astrocytes contribute to many CNS pathologies, little is known about the inflammatory pathways controlling gene expression. Our laboratory identified altered levels of tissue inhibitor of metalloproteinase (TIMP)-1 in brain lysates from human immunodeficiency virus (HIV)-1 infected patients, compared to age-matched controls, and interleukin (IL)-1β as a key regulator of astrocyte TIMP-1. Additionally, CCAAT enhancer binding protein (C/EBP)β levels are elevated in brain specimens from HIV-1 patients and the transcription factor contributes to astrocyte TIMP-1 expression. In this report we sought to identify key signaling pathways necessary for IL-1β-mediated astrocyte TIMP-1 expression and their interaction with C/EBPβ. Primary human astrocytes were cultured and treated with mitogen activated protein kinase-selective small molecule inhibitors, and IL-1β. TIMP-1 and C/EBPβ mRNA and protein expression were evaluated at 12 and 24 h post-treatment, respectively. TIMP-1 promoter-driven luciferase plasmids were used to evaluate TIMP-1 promoter activity in inhibitor-treated astrocytes. These data show that extracellular regulated kinase (ERK) 1/2-selective inhibitors block IL-1β-induced astrocyte TIMP-1 expression, but did not decrease C/EBPβ expression in parallel. The p38 kinase (p38K) inhibitors partially blocked both IL-1β-induced astrocyte TIMP-1 expression and C/EBPβ expression. The ERK1/2-selective inhibitor abrogated IL-1β-mediated increases in TIMP-1 promoter activity. Our data demonstrate that ERK1/2 activation is critical for IL-1β-mediated astrocyte TIMP-1 expression. ERK1/2-selective inhibition may elicit a compensatory response in the form of enhanced IL-1β-mediated astrocyte C/EBPβ expression, or, alternatively, ERK1/2 signaling may function to moderate IL-1β-mediated astrocyte C/EBPβ expression. Furthermore, p38K activation contributes to IL-1β-induced astrocyte TIMP-1 and C/EBPβ expression. These data suggest that ERK1/2 signals downstream of C/EBPβ to facilitate IL-1β-induced astrocyte TIMP-1 expression. Astrocyte ERK1/2 and p38K signaling may serve as therapeutic targets for manipulating CNS TIMP-1 and C/EBPβ levels, respectively.

## Introduction

Astrocytes are essential cells of the central nervous system (CNS) and are subject to the perturbations coinciding with neural pathologies, including human immunodeficiency virus (HIV)-1-associated neurocognitive disorders (HAND) [1], [2], [3]. During HAND, HIV-1-infected monocytes infiltrate the CNS where they disseminate viral particles, cytokines and other stimulatory molecules [4]. Cytokines and viral toxins produced in this inflamed environment may bring about deleterious changes in astrocyte gene expression [4], [5]. Dysfunctional astrocytes compromise optimal maintenance of the blood brain barrier, glutamate reuptake and the matrix metalloproteinase (MMP): tissue inhibitor of metalloproteinase (TIMP) balance [6], [7], [8], [9], [10], [11]. In the CNS astrocytes are major producers of TIMP-1 [5], [12], [13], a multifunctional glycoprotein that regulates extracellular matrix processing and cell growth/apoptosis [14], [15], [16]. TIMP-1 is expressed in multiple tissues, by various cell types and plays roles in angiogenesis, neurogenesis, metastasis and other physiological processes by binding MMPs to inhibit their function [17], [18], [19], [20]. TIMP-1 displays antiapoptotic activity independent of MMP-binding function; this phenomenon has led to a search for a definite TIMP-1 receptor [21]. TIMP-1 affects neuronal development by altering dendrite outgrowth [16]. These intriguing functions, along with TIMP-1 being the inducible form and highly prevalent in disease, are currently being studied in the context of cancer, ischemia, Alzheimer's disease and HIV-1-associated neurocognitive disorders (HAND) [17], [22], [23], [24].

Recent studies have expanded a diverse list of cell- and tissue-specific TIMP-1 functions [21], [25]. However, knowledge of specific signal transduction pathways regulating TIMP-1 remains scant and, where present, appears to depend upon the stimuli and expressing cell type. Transforming growth factor-β induces activator protein-1 (AP-1) to promote fibroblast TIMP-1 expression [26]. Histone deacetylase and extracellular regulated kinase (ERK) signaling may also be required for fibroblast TIMP-1 expression [27], [28]. ERK1/2 or p38 kinase (p38K), but not c-jun N-terminal kinase (JNK), are required for oncostatin M-induced murine fibroblast TIMP-1 expression [29]. In rat granulosa cells, protein kinase A-, p38K- and ERK1/2-selective inhibitors blocked human chorionic gonadotropin-induced TIMP-1 expression [30]. In the brain, TIMP-1 is regulated in a time- and cell-dependent manner [31]. Recent studies, using human astrocytes suggest that p38K pathway activity is required for maximal IL-1β-induced astrocyte TIMP-1 expression; however, p38K inhibition was not sufficient to totally block TIMP-1 production [32]. Expectedly, knockdown of the p65 unit of nuclear factor (NF)κB transcription machinery blocked IL-1β-induced astrocyte TIMP-1 [32]. Taken together, these data suggest p38K functions in conjunction with other signaling pathways during IL-1β-induced astrocyte TIMP-1 expression. Our group reported that CCAAT enhancer binding protein (C/EBP)β is expressed in brains of HIV-1 associated dementia (HAD) patients and contributes to regulating IL-1β-induced astrocyte TIMP-1 expression [33].

In the context of HIV-1 infection, TIMP-1 levels are reduced in HAD patients when compared with age-matched controls [5], [34]. Additionally, human astrocytes treated with inflammatory cytokines [interleukin (IL)-1β or tumor necrosis factor-α] initially demonstrated increased TIMP-1 expression; however, production regresses during chronic stimulation with IL-1β [34]. Identifying the key pathways initiating IL-1β-induced astrocyte TIMP-1 expression may provide a target for restoring CNS TIMP-1. In this study, we used a combination of small molecule mitogen activated protein kinase (MAPK)-selective inhibitors and IL-1β to explore the role of p38K and ERK1/2 signal transduction pathways in IL-1β-induced astrocyte TIMP-1 expression. We employed reporter and overexpression plasmids along with MAPK-selective inhibitors to study how the IL-1β pathway connects C/EBPβ to astrocyte TIMP-1 expression. Herein, we report ERK1/2 may provide a therapeutic target to restore CNS TIMP-1 levels. Furthermore, manipulating C/EBPβ expression, along with that of its binding partners, NFκB, AP-1, or others, may be a promising route to manipulating CNS TIMP-1 levels.

## Methods

### Isolation, cultivation, and activation of human astrocytes

Human astrocytes were isolated from first- and early second-trimester aborted specimens obtained from the Birth Defects Laboratory, University of Washington, Seattle, in full compliance with the ethical guidelines of the NIH. The institutional review boards of both the Universities of Washington and North Texas Health Science Center approved the collection of human tissues for research. The Birth Defects Laboratory obtained written consent from all tissue donors.

Astrocytes were isolated from specimens as originally described by Gardner [34]. Activation of astrocytes was achieved by applying IL-1β for various time intervals. IL-1β is a prototypical inflammatory cytokine expressed during HAD; making IL-1β an excellent choice for studying human astrocyte function during neuroinflammation [35]. We empirically tested IL-1β dosage to determine a concentration that promotes maximal activation of astrocytes, 20 ng/ml. In mouse models infected with IL-1β-expressing adenovirus, IL-1β levels reach 10 ng/mg total protein 7 days post-injection and a mean of 41 ng/mg throughout the striatum [36], [37]. Accordingly, these data provide relevant implications for astrocyte function in many pathologies involving neuroinflammation. All experimental conditions were analyzed in multiple replicates, and all results were confirmed in at least two astrocyte donors.

### Pharmacological inhibitors

Monolayers of astrocytes were treated with twice the final dose of the MAPK-selective inhibitors, SB203580, SB202190, U0126 and PD98059 (Santa Cruz Biotechnology, Santa Cruz, CA) for 1 h before IL-1β treatment (12 h hours for mRNA or 24 h for protein isolation). Multiple doses of SB203580 (sc-353), and U0126 (sc-202374, 0.1-20 µM), and a single dose of SB202190 (sc-222294, 20 µM) and PD98059 (sc-3532, 20 µM) were tested as described in similar signaling studies [38], [39], [40], [41].

### RNA Isolation and real-time reverse transcription polymerase chain reaction (RT^2^PCR)

Astrocyte RNA was extracted (RNeasy plus mini kit, Qiagen, Alameda, CA) and reverse-transcribed into cDNA as per the manufacturer's instructions (High Capacity cDNA Reverse Transcription Kit, Life Technologies Corp., Carlsbad, CA). TaqMan® 5' nuclease RT^2^PCR assays were performed using a StepOnePlus system (Life Tech). The following TaqMan® gene expression assay primers were used: TIMP-1 (Life Tech, C/N: Hs99999139_m1), C/EBPβ (C/N: Hs00270923_s1) and glyceraldehyde phosphate dehydrogenase (GAPDH; C/N: 4310859). The reactions were carried out at 48°C for 30 min, 95°C for 10 min, followed by 40 cycles of 95°C for 15 s and 60°C for 1 min. Transcripts were quantified by the comparative C_T_ method, and are represented as fold-change of control [42]. Samples were analyzed in triplicate.

### Western Blot

Astrocytes were cultured as adherent monolayers in 75 cm^2^ flasks at a density of 8×10^6^ cells per flask. The following day, cells were treated with MAPK-selective inhibitors (SB203580 and U0126) for one h and then IL-1β (20 ng/ml) for 24 h. Alternately, for western blot detection of protein phosphorylation status, astrocytes were activated for 30 min with IL-1β. Cells were lysed; total cell extracts were isolated using mammalian protein extraction reagent (Thermo Fisher Scientific, Waltham, MA, USA) or nuclear extracts were isolated using nuclear extraction reagent (Sigma-Aldrich Inc., St. Louis, MO). Equal amounts of protein (15 µg/lane) were resolved by 12% sodium dodecyl sulfate polyacrylamide gel electrophoresis (SDS-PAGE) and subsequently transferred to a polyvinyl diflouride membrane using i-Blot (Life Tech). The membrane was incubated in anti-C/EBPβ (C-19, Santa Cruz), p38K, phosphorylated (P)-p38K, ERK and P-ERK1/2 at a dilution of 1∶200 (Cell Signaling Inc., Boston, MA, USA). Blots were then incubated in secondary antibody at a dilution of 1∶5,000. β-actin (Sigma) or lamin A/C (Cell Signaling), as a loading control. The western blot was visualized with supersignal chemiluminescent substrate (Thermo Fisher) and band intensities were quantified by densitometry analysis (ProteinSimple, Santa Clara, CA, USA).

### Measurement of TIMP-1 protein and 3-(4,5-dimethylthiazol-2-yl)-2,5-diphenyltetrazolium bromide (MTT) assay

TIMP-1 levels in astrocyte supernatants were measured using a commercially available enzyme-linked immunosorbent assay (ELISA) kit (R&D Systems, Minneapolis, MN). The MTT assay was performed at appropriate time points according to the method originally described by Manthrope [43]. The ELISA determinations yielded quantities of protein in units of ng/ml and were normalized to MTT values.

### Transfection of primary human astrocytes with luciferase reporter constructs

Three TIMP-1-luc promoter plasmid (pTIMP-1-luc) constructs were used to measure TIMP-1 promoter activity in transfected astrocytes. The −4200/+96 and −1718/+96 portions of the TIMP-1 sequence were cloned into the pGL3-basic reporter vector (Promega, Madison, WI) and were kindly provided by Dr. Ian Clark at the University of East Anglia, UK [44].

Astrocytes were cultured as adherent monolayers in a 48-well plate at a density of 0.15×10^6^ cells per well. The following day, cells were transfected with 1.5 µg of total DNA using Lipofectamine™ reagent as per the manufacturer's instructions (Life Tech). Total DNA consisted of a mixture of the pTIMP-1-luc and the pGL3-driven Renilla luciferase (pGL3-RL, Promega) plasmids. Twenty-four h post-transfection media was aspirated from selected wells and replaced with media supplemented with MAPK-selective inhibitors at 20 µM for 24 h with or without 20 ng/ml IL-1β. At two days post-transfection promoter activity was measured as luciferase activity in cell extracts using the Dual-Glo® luciferase assay system per the manufacturers instructions (Promega). Luciferase activity was determined as a ratio of firefly to Renilla luciferase using the a Glomax luminimeter (Promega). Renilla luciferase activity was used as an internal control. All experimental conditions were analyzed in multiple replicates, and all results were confirmed in at least two astrocyte donors.

### Immunocytochemistry

Astrocytes were cultured as adherent monolayers in a 48-well plate at a density of 0.1×10^6^ cells per well. The following day, cells were treated with 20 µM concentration of SB203580 or U0126 for 1 h and then with IL-1β for 24 h. Cells were fixed with cold acetone∶methanol (1∶1) for 30 mins at −20°C. Cells were then blocked in phosphate buffered saline (PBS) with 2% bovine serum albumin for 1 h at room temperature. Cells were incubated in PBS (2% bovine serum albumin, 0.1% triton X) plus C/EBPβantibody at 1∶500 and glial fibrillary acidic protein (GFAP) at 1∶600 for 8 h at 4°C. Cells were washed with PBS and then incubated with Alexa Fluor secondary antibody (Life Tech, 1∶800) for 1 h at room temperature. Micrographs were taken on a Nikon Eclipse T*_i_*. (Nikon Inc., Melville, NY, USA).

### Statistical Analyses

Results were analyzed with GraphPad Prism 5.0 (GraphPad Software, Inc., La Jolla, CA) using one-way analysis of variance with Newman Keul's post-test for multiple comparisons. Significance was set at p<0.05 and data represent mean values ± standard error of the mean. Data presented are representative of a minimum of three independent experiments with two or more independent donors. In all figures, statistical significance is represented as ^*^p<0.05, ^**^p<0.01, and ^***^p<0.001, significance indicates versus control unless indicated by bar.

## Results

### p38K- and ERK1/2-selective inhibitors block IL-1β-mediated primary human astrocyte TIMP-1 expression

**Figure 1:**
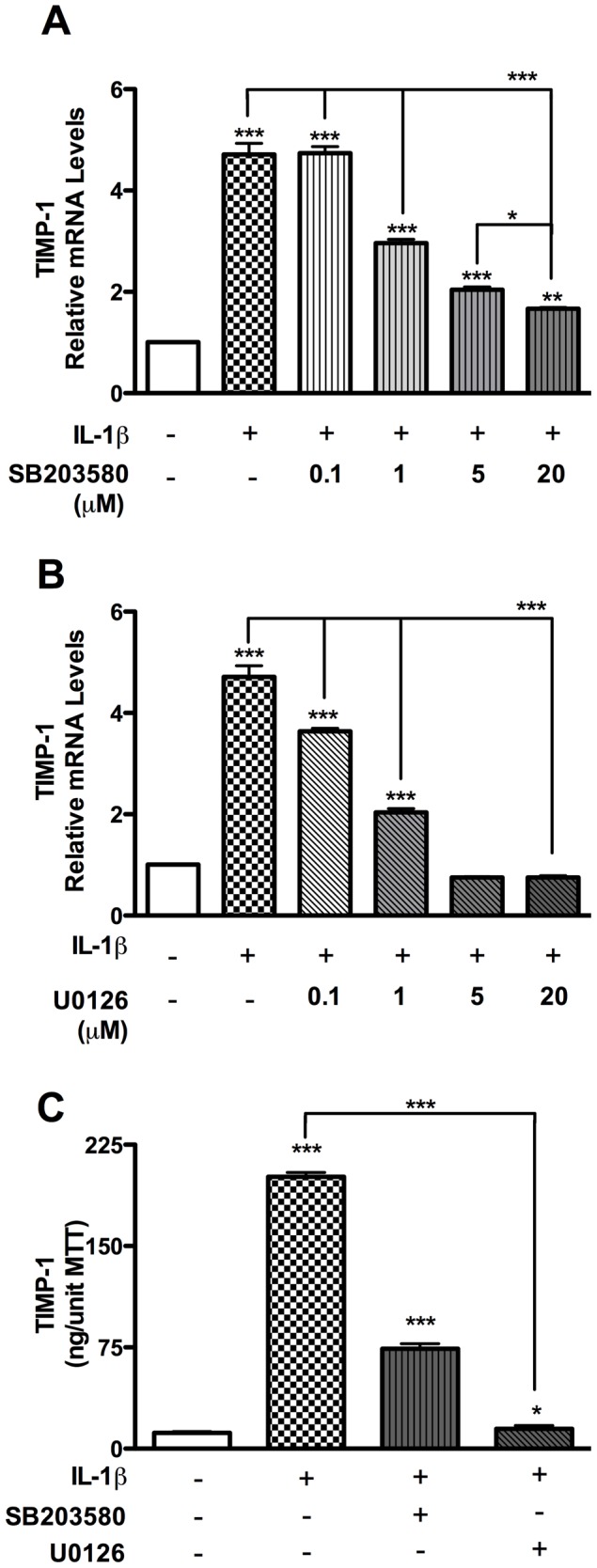
p38K- and ERK1/2-selective small molecule inhibitors block IL-1β-mediated astrocyte TIMP-1. (**A-B**) Astrocytes were treated with increasing doses of p38K- and ERK1/2-selective inhibitors (SB203580 and U0126, respectively) for 1 h, and then IL-1β (20 ng/ml) for 12 h. Total RNA was collected and reverse transcribed. TIMP-1 and GAPDH transcripts were quantified by real-time PCR. (**C**) In parallel, supernatants from 48 well cultures were collected after 24 h IL-1β treatment. Supernatants were analyzed for TIMP-1 expression by ELISA and normalized via MTT assay. Data presented are representative of a minimum of three independent experiments with two or more independent donors. (^*^p<0.05, ^**^p<0.01, ^***^p<0.001; significance indicates versus untreated unless indicated by bar).

Reports suggest that p38K is involved in IL-1β-induced astrocyte TIMP-1 expression, but the role of the ERK1/2 signaling pathway remains unexplored [32]. In the following studies we tested the efficacy of increasing doses of p38K- and ERK1/2-selective inhibitors in blocking IL-1β-induced astrocyte TIMP-1 expression. Pretreatment with SB203580 or U0126 prevented IL-1β-induced p38K and ERK1/2 phosphorylation, respectively (Figure S1). Consistent with previous findings [5], [11], IL-1β led to a 5-fold increase in TIMP-1 mRNA levels 12 h post-treatment (p<0.001, Figure 1A and B). TIMP-1 mRNA levels were elevated in astrocytes treated with increasing doses of SB203580; however, 1, 5 and 20 µM concentrations of the inhibitor reduced the TIMP-1 response by 40, 60 and 65%, respectively (p<0.001, Figure 1A). IL-1β-induced astroctye TIMP-1 mRNA levels were reduced by 25% in astrocytes treated 0.1 µM U0126, 60% in astrocytes treated with 1 µM U0126, and 5 µM U0126 resulted in basal TIMP-1 levels (p<0.001, Figure 1B). Analysis of cell supernatants, 24 h post treatment, showed that TIMP-1 protein production was statistically significantly greater (∼200-fold) in cells treated with IL-1β as compared to control cells (Figure 1C). Furthermore, p38K- and ERK1/2-selective inhibitors significantly reduced TIMP-1 protein levels in IL-1β-treated cells (SB203580: 3-fold versus IL-1β, p<0.001, and U0126: 13-fold versus IL-1β, p<0.05, Figure 1C); with U0126 exhibiting the highest quantitative change both at mRNA and at protein level. Two additional p38K- and ERK1/2-selective inhibitors, SB202190 and PD98059, were also effective at inhibiting IL-1β-induced astrocyte TIMP-1 expression (Figure S2). These data suggest that p38K-selective inhibitors affect IL-1β-induced astrocyte TIMP-1 expression; however, the ERK1/2-selective inhibitor is most effective.

### p38K and ERK1/2 pathways differentially regulate C/EBPβ mRNA and nuclear protein levels

**Figure 2:**
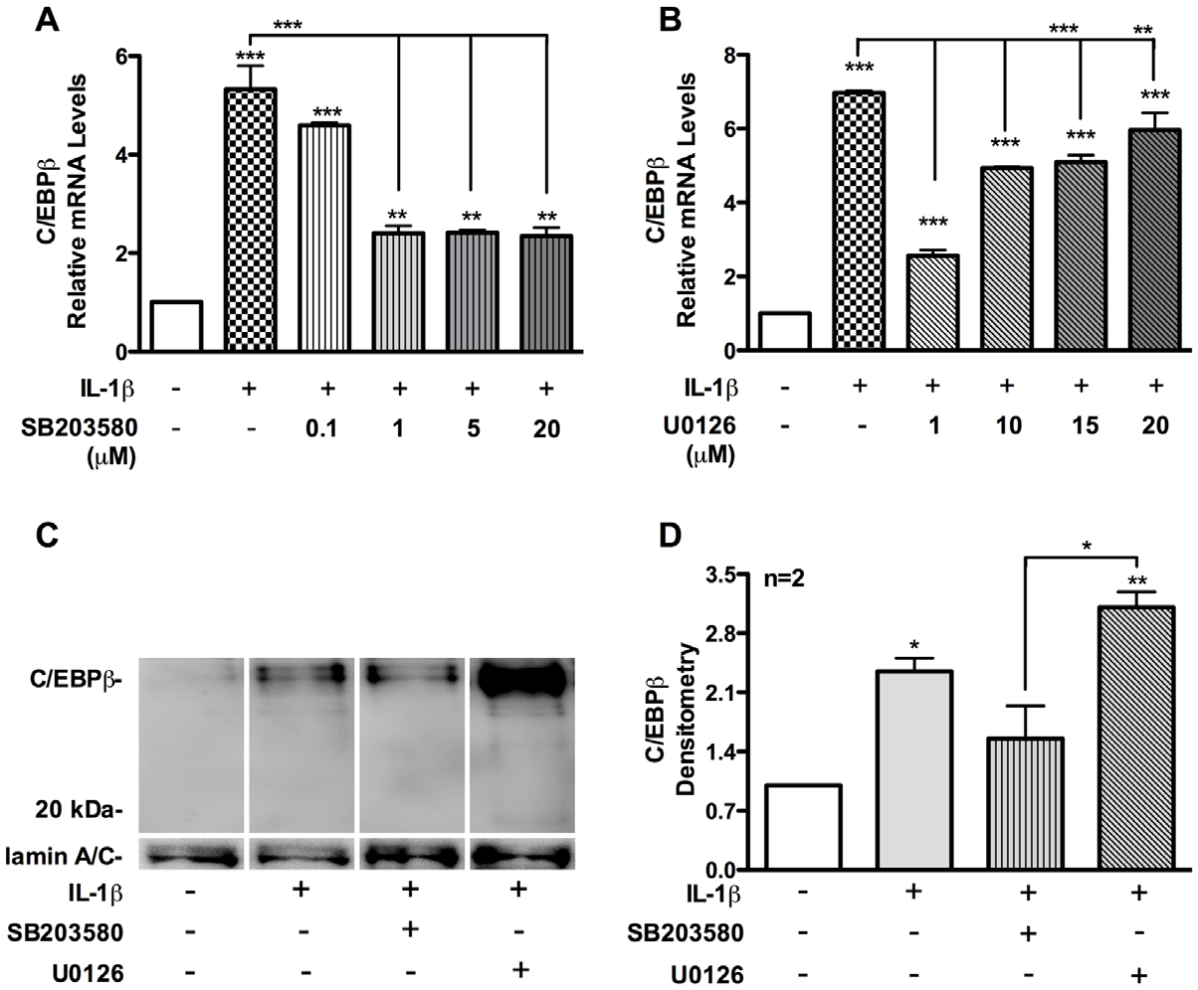
p38K- and ERK1/2-selective small molecule inhibitors block and enhance IL-1β-mediated astrocyte C/EBPβ expression, respectively. (A-B) Astrocytes were treated with increasing doses of p38K- and ERK1/2-selective inhibitors (SB203580 and U0126, respectively) for 1 h, and then IL-1β (20 ng/ml) for 12 h. Total RNA was collected and reverse transcribed. C/EBPβ and GAPDH transcripts were quantified by real-time PCR. (C) Nuclear extracts were isolated 24 h post-IL-1β treatment, resolved by SDS-PAGE and then immunoblotted for C/EBPβ and lamin A/C protein. (D) C/EBPβ and lamin A/C band intensity was analyzed by densitometry analysis. Data presented are representative of a minimum of three independent experiments with two or more independent donors (^*^p<0.05, ^**^p<0.01, ^***^p<0.001; significance indicates versus untreated unless indicated by bar).

Previously, we reported that C/EBPβ is expressed in brain lysates of HIV-1-infected individuals, and that C/EBPβ contributes to IL-1β-induced astrocyte TIMP-1 expression [33]. C/EBPβ knockdown partially blocked IL-1β-induced astrocyte TIMP-1 expression; however, blocking ERK1/2 activation resulted in >90% reduction in IL-1β-induced astrocyte TIMP-1 expression (Figure 1B). These studies suggest that ERK1/2 is a critical pathway for IL-1β-induced astrocyte TIMP-1 expression. In the current studies, we aimed to elucidate the signal transduction pathways controlling IL-1β-induced astrocyte C/EBPβ expression. IL-1β induced astrocyte C/EBPβ mRNA levels by ∼5-fold compared to control (p<0.001, Figure 2A). The p38K-selective inhibitor, SB203580, showed a dose-dependent effect on IL-1β-induced astrocyte C/EBPβ mRNA expression. SB203580 at 0.1 µM reduced IL-1β-induced astrocyte C/EBPβ mRNA expression by 15%, and by ∼55% at concentrations >1 µM (p<0.001, Figure 2A). IL-1β induced astrocyte C/EBPβ mRNA expression at doses of SB203580 through 20 µM. A low 1 µM dose of U0126 reduced IL-1β-induced astrocyte C/EBPβ mRNA expression but had lesser inhibitory effects at higher doses 10–20 µM (p<0.001, Figure 2B). Two additional p38K- and ERK1/2-selective inhibitors, SB202190 and PD98059, were used to assess their effectiveness at inhibiting IL-1β-induced C/EBPβ mRNA expression (Figure S2). The p38K inhibitor (SB202190) inhibited IL-1β-induced astrocyte C/EBPβ mRNA expression; however, the ERK1/2 inhibitor (PD98059) increased the expression of C/EBPβ during inflammation.

C/EBPβ isoforms were undetectable in nuclear lysates from control astrocytes; however, increased C/EBPβ, 42 and 40 kDa isoforms, were detected in IL-1β-treated astrocytes (Figure 2C). C/EBPβ isoforms were decreased in SB203580-treated astrocytes, but increased in U0126-treated astrocytes (Figure 2C). C/EBPβ band intensity from IL-1β-treated astrocytes was increased by 2.5-fold compared to control cells (p<0.05, Figure 2D). IL-1β-induced C/EBPβ band intensity from SB203580-treated astrocytes was increased by 1.5-fold compared to control cells, whereas IL-1β-induced C/EBPβ band intensity from U0126-treated astrocytes was increased by 3-fold compared to control astrocytes (p<0.001; Figure 2B). These data were corroborated in experiments using additional p38K- and ERK1/2-selective inhibitors, each of which produced similar results at the mRNA level (Figure S2). Overall, these data show that p38K activation is required for full IL-1β-induced astrocyte TIMP-1 and C/EBPβ expression. ERK1/2 activation is critical for IL-1β-induced astrocyte TIMP-1 expression; however, ERK1/2 inhibition may enhance IL-1β-induced astrocyte C/EBPβ expression.

**Figure 3:**
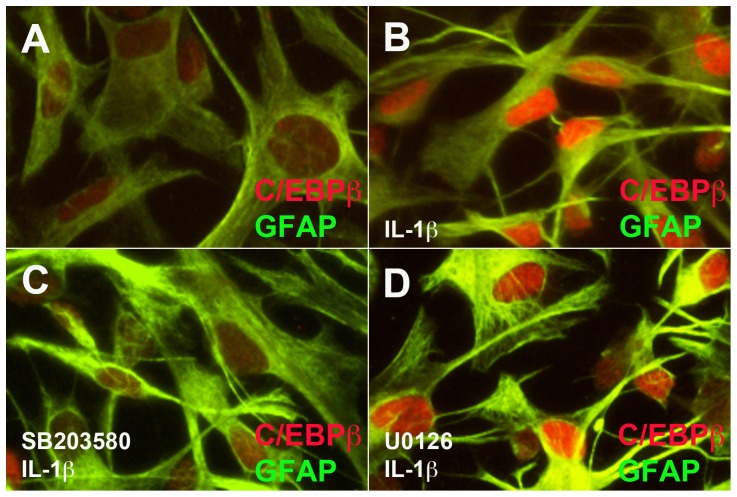
p38K-selective small molecule inhibitor blocks C/EBPβ localization to the nucleus in response to IL-1β. (**A-D**) Astrocytes were cultured in 48-well plates for 24 h, treated with selective inhibitors (SB203580, 20 µM and U0126, 20 µM) for 1 h, and then IL-1β (20 ng/ml) for 24 h. Cells were fixed, blocked and then incubated in antibodies against GFAP (green) and C/EBPβ (red). All pictures were taken at X200 and data represent at least three independent experiments in at least two independent donors.

As a transcription factor, C/EBPβ is localized to nuclei where it can affect gene transcription. We pretreated astrocytes with inhibitors and then IL-1β for 24 h, fixed and colocalized GFAP as an astrocyte-specific marker (green) with C/EBPβ (red) in control and activated human astrocytes. In control cells, GFAP is present throughout the cell body of the astrocytes. Control astrocytes have a larger cell body (Figure 3A), whereas activated astrocytes have extensive processes protruding from their soma and denser staining of GFAP (Figure 3B-D). Low levels of C/EBPβ are present in the nuclei of control human astrocytes (Figure 3A) compared with activated astrocytes (Figure 3B), where the red signal from the nuclei is markedly enhanced. Low levels of C/EBPβ are present in the nuclei of SB203580-pretreated astrocytes (Figure 3C) compared to those treated with IL-1β alone (Figure 3B) or U0126-pretreated astrocytes (Figure 3D). Intense red signal is detected in nuclei of U0126-pretreated astrocytes (Figure 3D). Combined with the mRNA and protein expression data represented earlier, these data suggest that blocking p38K activation decreases IL-1β-induced astrocyte C/EBPβ nuclear expression.

### ERK1/2-selective inhibitor, U0126, overrides IL-1β- or C/EBPβ-mediated increase in TIMP-1 promoter activity

**Figure 4:**
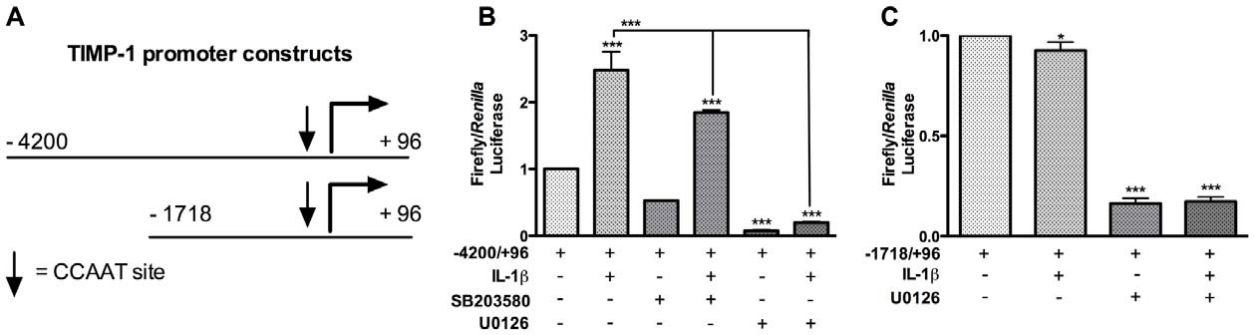
ERK1/2-selective inhibitor, U0126, overrides IL-1β- and C/EBPβ-mediated increases in TIMP-1 promoter activity. Astrocytes were cultured in 48-well plates for 24 h and then cotransfected with TIMP-1 promoter-driven luciferase plasmids (**B**, −4200/+96 or **C** −1718/+96) and internal control pGL3-RL. (**A**) The −4200/+96 promoter harbors an IL-1β responsive element, while the −1718/+96 is the basal TIMP-1 promoter. Both promoters possess a predicted CCAAT-binding site prior to the start site [44]. Cells were allowed to recover for 24 h and then treated with MAPK-selective inhibitors (SB203580, 20 µM; and U0126, 20 µM) for 1 h prior to activation with IL-1β (20 ng/ml) for 24 h. Total cell lysates were collected 24 h post-inhibitor treatment and assayed for luciferase expression. Data represent mean values ± standard error of the mean in at least three independent experiments in at least two independent donors (^*^p<0.05, ^**^p<0.01, ^***^p<0.001; significance indicates versus untreated unless indicated by bar).

We previously demonstrated that overexpressing C/EBPβ increases transcription from −1718/+988 of the TIMP-1 promoter [33]. Here we used −4200/+96or −1718/+96 driven-pTIMP-luc expression in combination with selective inhibitors and IL-1β to investigate regulation of TIMP-1 transcription. The two TIMP-1 promoter regions, −4200/+96 and−1718/+96, each contain the CCAAT site near the transcription start site. However, the −4200/+96 TIMP-1 promoter region retains the IL-1β-responsive element, while the −1718/+96 TIMP-1 promoter region has the basal TIMP-1 promoter (Figure 4A)

Astrocytes were cotransfected with pTIMP-luc and pGL3-RL and then treated with selective inhibitors and/or IL-1β. IL-1β induced a 2.5-fold (p<0.001) increase in luciferase activity from the −4200/+96 region, but U0126 treatment completely blocked this increase, as well as basal promoter activity (p<0.001, Figure 4B). SB203580 treatment reduced the IL-1β-mediated effect by 25% (p<0.001) (Figure 4B). These data corroborate our results from experiments using mRNA and protein assays. To better understand the relationship between ERK1/2 and C/EBPβ we assayed a promoter, lacking the IL-1β-responsive element, −1718/+96. IL-1β significantly reduced transcription from −1718/+96-driven pTIMP-luc, and U0126 treatment extended this reduction (p<0.05 and p<0.001, respectively; Figure 4C). These data suggest that ERK1/2 signaling is required for IL-1β- mediated transcription from −4200/+96 of the TIMP-1 promoter.

## Discussion

Currently many studies focus on TIMP-1 activity in many diseases, multiple tissues and cell types [16], [17], [45]; however, the regulation of TIMP-1 expression is as important as its activity in the tissue microenvironment. As previously mentioned, data suggest multiple signal transduction pathways may regulate TIMP-1 expression depending upon stimuli and the expressing cell [46]. In these studies we aimed to identify signaling pathways responsible for IL-1β-induced astrocyte TIMP-1 expression. Our data indicate, for the first time, that the ERK1/2 pathway is essential for IL-1β-mediated increases in astrocyte TIMP-1 and ERK1/2-targeted pharmacological inhibitors will prove sufficient in abrogating astrocyte-TIMP-1 upregulation. In these studies, we show that the p38K-selective inhibitors partially, but significantly, reduce IL-1β-mediated increases in astrocyte TIMP-1 mRNA in a dose-dependent manner up to 1 µM; at which point, it plateaus. p38K-selective inhibitors also reduced IL-1β-induced astrocyte TIMP-1 protein, suggesting the p38K pathway contributes to IL-1β-mediated increases in astrocyte TIMP-1. Concentrations of ERK1/2-selective inhibitor, U0126, as low as 0.1 µM significantly reduced IL-1β-mediated increases in astrocyte TIMP-1; however, 5–20 µM concentrations reduced TIMP-1 mRNA below basal levels. Inhibiting the ERK1/2 pathway is sufficient to block IL-1β-mediated increases in astrocyte TIMP-1 protein, indicating that the ERK1/2 pathway is critical for IL-1β-mediated increases in astrocyte TIMP-1. ERK1/2-selective inhibitors were most effective at blocking IL-1β-induced astrocyte TIMP-1 expression, at all doses tested. Blocking p38K activity diminishes IL-1β-mediated increases in astrocyte C/EBPβ. Interestingly, blocking ERK1/2 activation with 20 µM of U0126 may enhance IL-1β-induced C/EBPβ expression while at the same time blocking IL-1β-mediated increases in astrocyte TIMP-1 expression. Lastly, blocking ERK1/2 activation was shown to block IL-1β- or C/EBPβ-mediated increases in TIMP-1 promoter activity, suggesting ERK1/2 signals to key transcription factors necessary for C/EBPβ-mediated TIMP-1 promoter regulation. These data may shed light on how astrocytes regulate C/EBPβ and TIMP-1 expression during neuroinflammation by identifying essential and auxiliary pathways.

Inflammatory stimuli lead to rapid phosphorylation of MAPK, p38K and ERK1/2 in astrocytes, initiating a cascade of signal transduction events that ultimately influence cellular function [47]. ERK1/2 and p38K regulate astrocyte morphology, cell death and extracellular matrix turnover [38], [39], [40]. Furthermore, small molecule inhibitors of signal transduction pathways are being explored as treatment options for multiple diseases [41]. At minimum, small molecule inhibitors are useful tools to identify pathways responsible for cellular processes [48]. The p38K-selective inhibitor, SB203580, inhibited IL-1β-mediated increases in astrocyte TIMP-1 mRNA by 50% at doses as low as 5 µM and as high as 20 µM. Pretreatment with the 0.1-20 µM concentrations of ERK1/2-selective inhibitor, U0126, reduced IL-1β-mediated increases in astrocyte TIMP-1 mRNA expression to basal levels. Additional p38K- (SB202190) and ERK1/2- (PD98059) selective inhibitors were similarly able to block IL-1β-mediated increases in astrocyte TIMP-1 expression (Figure S2). Crosstalk among MAPK proteins may account for multiple MAPK-selective inhibitors' effect on IL-1β-mediated increases in astrocyte TIMP-1. Alternatively p38K and ERK1/2 may phosphorylate, to different degrees, the same or different transcription factors that bind to astrocyte TIMP-1 promoter. In either case, we add TIMP-1, a multifunctional molecule of major importance to CNS health and disease, to the list of astrocyte genes critically regulated by the ERK1/2 pathway.

It is well established that NFκB mediates IL-1β-induced gene expression in many cell types, including astrocytes. However, NFκB activity alone may not account for the massive changes in gene expression following immune stimulation; indeed, p65 and p50 associate with multiple factors, including C/EBP's, to regulate gene transcription[49]. Wilczynska *et al*. showed that NFκB is involved in IL-1β-induced astrocyte TIMP-1 expression [32]. Here, we focused on elucidating the role of another transcription factor, C/EBPβ that is expressed in astrocytes and is associated with inflammatory responses. C/EBPβ is expressed in the brain of HIV-1-infected individuals and contributes to IL-1β-mediated increases in astrocyte TIMP-1 [33]. Therefore, it was surprising that ERK1/2-selective inhibitors did not reduce and may enhance IL-1β-mediated increases in astrocyte C/EBPβ expression while decreasing TIMP-1 expression. p38K-selective inhibitor, SB203580, decreased IL-1β-mediated increases in astrocyte C/EBPβ and TIMP-1 expression. The fact that ERK1/2 inhibition strongly blocks IL-1β-mediated increases in astrocyte TIMP-1 suggests ERK1/2 may activate one or more critical transcription factors that lay the framework for IL-1β-mediated increases in astrocyte TIMP-1, independent of C/EBPβ activity. Consistent with previous reports, IL-1β induces transcription from the TIMP-1 promoter region containing the upstream elements between −2200 and −2700 [32]. However, ERK1/2 inhibition was more effective than p38K inhibition at blocking IL-1β-induced astrocyte TIMP-1 expression; suggesting ERK1/2 may activate factors that bind the TIMP-1 promoter IL-1β-responsive element. Data suggest that ERK1/2 signaling can repress C/EBPβ activity through direct phosphorylation [49]. In control human astrocytes ERK1/2 activity may affect C/EBPβ activity indirectly through regulation of binding partners. C/EBPβ may utilize some, or all, of the TIMP-1 basal promoter, exon 1 and 2 and five CCAAT sites [44]. Our data show that IL-1β induces C/EBPβ nuclear localization in U0126-pretreated cells; suggesting other critical factors required for C/EBPβ increases in TIMP-1 promoter activity may be absent in these conditions. These data suggest that ERK1/2 activity is required for IL-1β- and C/EBPβ-mediated increases in astrocyte TIMP-1 expression.

## Conclusions

**Figure 5:**
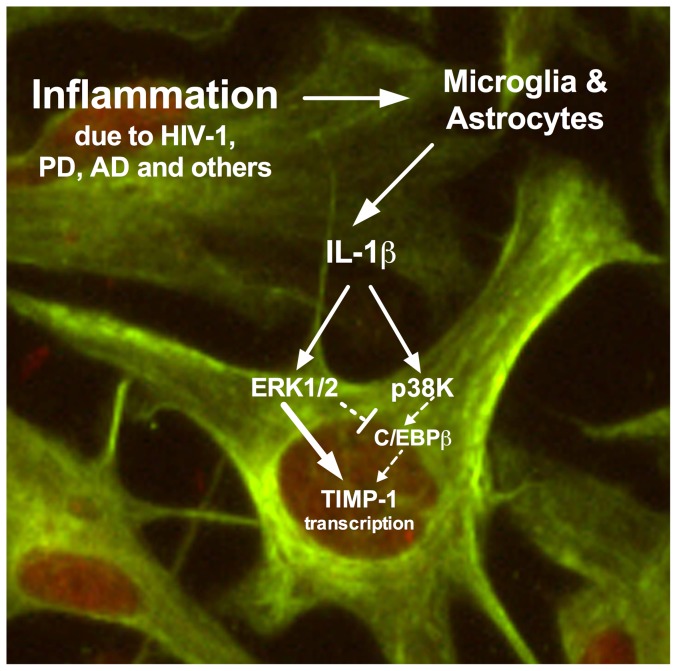
ERK1/2 is the predominant signaling pathway facilitating IL-1β-mediated astrocyte TIMP-1 expression. Neuroinflammation activates bystander microglia and astrocytes to secrete IL-1β. Astrocytes respond to IL-1β by secreting TIMP-1. In this study we use a panel of small molecule pathway-selective inhibitors to delineate the signal transduction mechanism facilitating IL-1β-induced astrocyte TIMP-1 expression. p38K- and ERK1/2-selective inhibitors reduced and completely blocked IL-1β-induced astrocyte TIMP-1 mRNA and protein expression, respectively. U0126, the ERK1/2-selective inhibitor, also blocked TIMP-1 promoter activity.

TIMP-1 expression has been studied in several cell types, but astrocyte TIMP-1 regulation is not completely understood [14], [24], [50]. Wilczynska *et al*. showed NFκB and p38K are involved in IL-1β-induced astrocyte TIMP-1 expression; however, our studies suggest, though p38K may be involved, ERK1/2 is critical for basal and IL-1β-activated astrocyte TIMP-1 expression (Figure 5) [32]. Recently it was reported that TIMP-1 activates the AKT pathway in fibroblasts, indicating the molecule initiates further cascading of signal transduction, presumably playing an important role in response to inflammation [51]. Given the multiple functions of TIMP-1, restoring TIMP-1 levels in HAND patients may prevent or reverse deleterious effects of an MMP:TIMP imbalance and TIMP-1 deficiency. Knowledge of astrocyte TIMP-1 regulation may lead to identification of novel pharmacological targets, and ultimately therapies for HAND patients.

## Supporting Information

Figure S1 **MAPK-selective inhibitors block IL-1β-induced astrocyte p38K and ERK1/2 phosphorylation.** (**A and C**) Astrocytes were treated with of p38K- and ERK1/2-selective inhibitors (SB203580 and U0126, respectively) for 1 h, and then IL-1β (20 ng/ml). Total protein extracts were isolated 30 min post-IL-1β treatment, resolved by SDS-PAGE and then immunoblotted for p38K, P-p38K, ERK/2 and P-ERK1/2. (**B and D**) Phosphorylated and nonphosphorylated isoforms' band intensity was analyzed by densitometry analysis. Data presented are representative of a minimum of three independent experiments with two independent donors (n = 2), (^*^p<0.05; significance indicates versus untreated unless indicated by bar).
(TIF)

Figure S2 **p38K- and ERK1/2-selective small molecule inhibitors alter IL-1β-mediated astrocyte TIMP-1 and C/EBPβ expression.** (**A and C**) Astrocytes were treated with the p38K-selective inhibitor, SB202190, for 1 h, and then IL-1β (20 ng/ml) for 12 h. Total RNA was collected and reverse transcribed. TIMP-1, C/EBPβ and GAPDH transcripts were quantified by real-time PCR. (**B and D**) Astrocytes were pretreated with the ERK1/2-selective inhibitor, PD98059, for 1 h, and then IL-1β (20 ng/ml) for 12 h. Total RNA was collected and reverse transcribed. TIMP-1, C/EBPβ and GAPDH transcripts were quantified by real-time PCR. Data presented are representative of a minimum of three independent experiments with two or more independent donors (^*^p<0.05, ^***^p<0.001; significance indicates versus untreated unless indicated by bar).
(TIF)
