# Supplementary figures and images for: Extracellular Regulated Kinase 1/2 Signaling Is a Critical Regulator of Interleukin-1β-Mediated Astrocyte Tissue Inhibitor of Metalloproteinase-1 Expression
Source: PLoS One. 2013 Feb 14;8(2):e56891. doi: 10.1371/journal.pone.0056891 (PMC3572966; doi:10.1371/journal.pone.0056891)

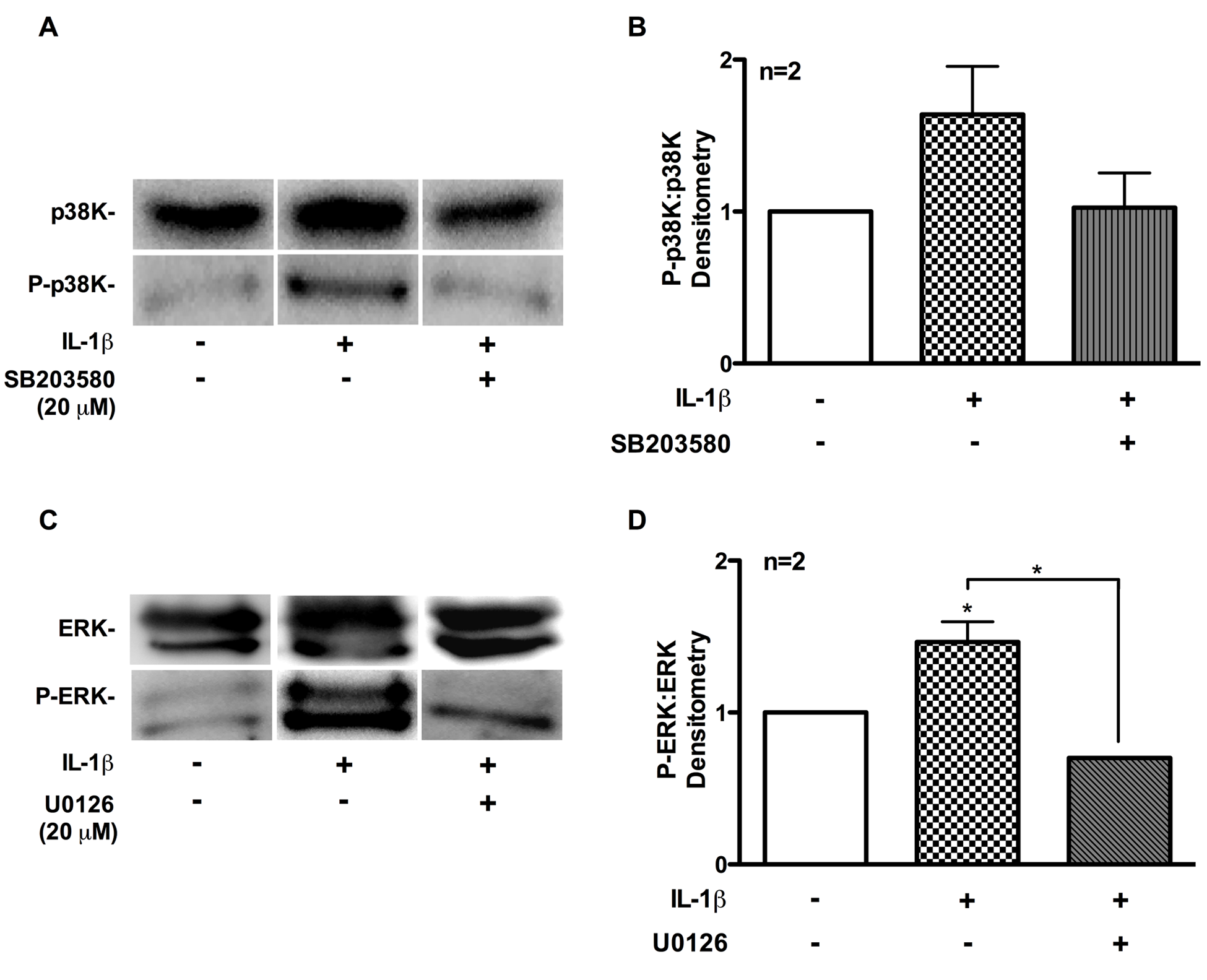

Supplement: Figure S1 — MAPK-selective inhibitors block IL-1β-induced astrocyte p38K and ERK1/2 phosphorylation. (A and C) Astrocytes were treated with of p38K- and ERK1/2-selective inhibitors (SB203580 and U0126, respectively) for 1 h, and then IL-1β (20 ng/ml). Total protein extracts were isolated 30 min post-IL-1β treatment, resolved by SDS-PAGE and then immunoblotted for p38K, P-p38K, ERK/2 and P-ERK1/2. (B and D) Phosphorylated and nonphosphorylated isoforms' band intensity was analyzed by densitometry analysis. Data presented are representative of a minimum of three independent experiments with two independent donors (n = 2), (*p<0.05; significance indicates versus untreated unless indicated by bar). (TIF) [file pone.0056891.s001.tif]

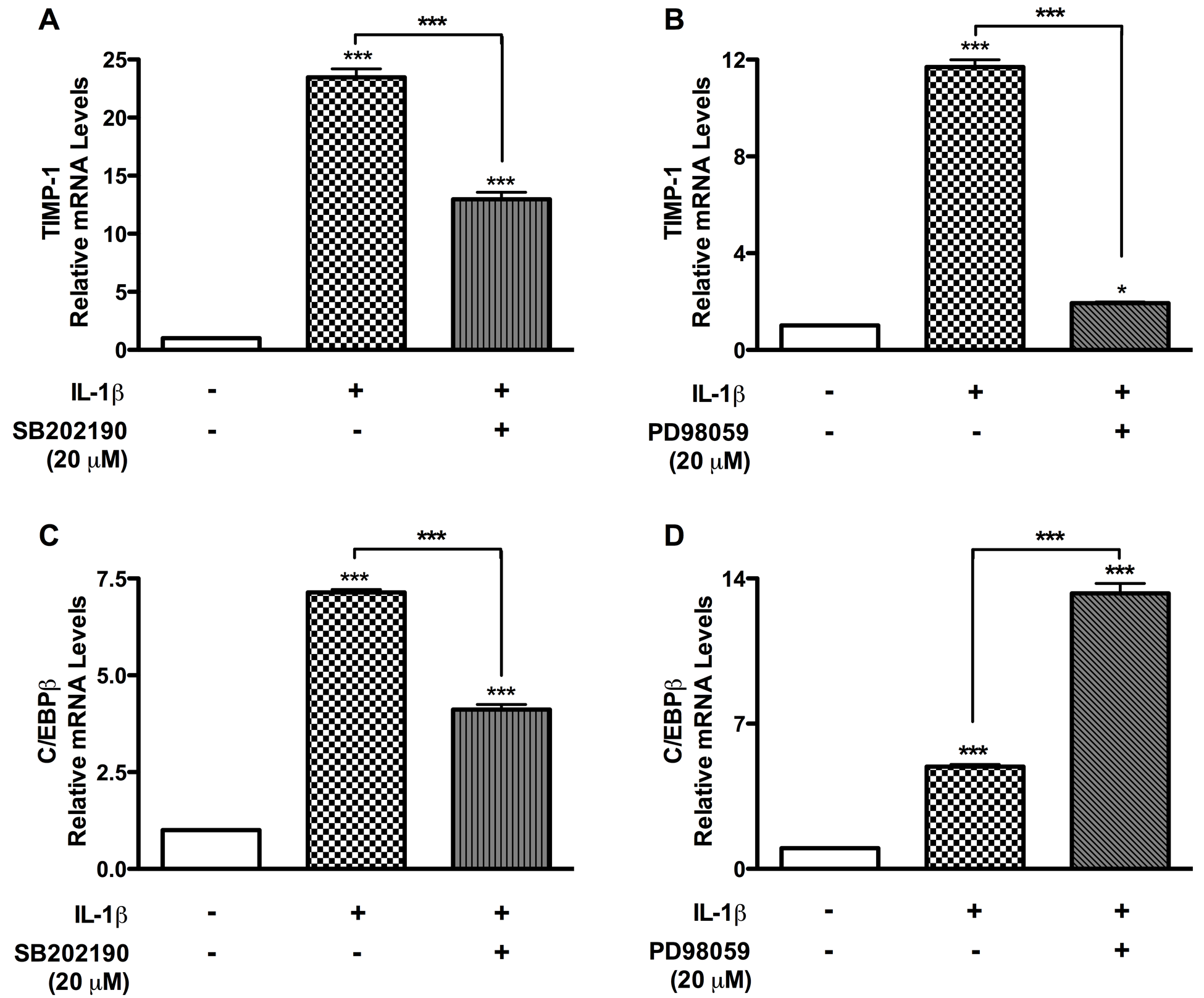

Supplement: Figure S2 — p38K- and ERK1/2-selective small molecule inhibitors alter IL-1β-mediated astrocyte TIMP-1 and C/EBPβ expression. (A and C) Astrocytes were treated with the p38K-selective inhibitor, SB202190, for 1 h, and then IL-1β (20 ng/ml) for 12 h. Total RNA was collected and reverse transcribed. TIMP-1, C/EBPβ and GAPDH transcripts were quantified by real-time PCR. (B and D) Astrocytes were pretreated with the ERK1/2-selective inhibitor, PD98059, for 1 h, and then IL-1β (20 ng/ml) for 12 h. Total RNA was collected and reverse transcribed. TIMP-1, C/EBPβ and GAPDH transcripts were quantified by real-time PCR. Data presented are representative of a minimum of three independent experiments with two or more independent donors (*p<0.05, ***p<0.001; significance indicates versus untreated unless indicated by bar). (TIF) [file pone.0056891.s002.tif]
